# Impact of race on efficacy and safety during treatment with olanzapine in schizophrenia, schizophreniform or schizoaffective disorder

**DOI:** 10.1186/1471-244X-10-89

**Published:** 2010-11-03

**Authors:** Virginia L Stauffer, Jennifer L Sniadecki, Kevin W Piezer, Jennifer Gatz, Sara Kollack-Walker, Vicki Poole Hoffmann, Robert Conley, Todd Durell

**Affiliations:** 1 Lilly USA, LLC, Lilly Corporate Center, Indianapolis, IN 46285, USA

## Abstract

**Background:**

To examine potential differences in efficacy and safety of treatment with olanzapine in patients with schizophrenia of white and black descent.

**Methods:**

A post-hoc, pooled analysis of 6 randomized, double-blind trials in the treatment of schizophrenia, schizophreniform disorder, or schizoaffective disorder compared white (N = 605) and black (N = 375) patients treated with olanzapine (5 to 20 mg/day) for 24 to 28 weeks. Efficacy measurements included the Positive and Negative Syndrome Scale (PANSS) total score; and positive, negative, and general psychopathology scores; and the Clinical Global Impression of Severity (CGI-S) scores at 6 months. Safety measures included differences in the frequencies of adverse events along with measures of extrapyramidal symptoms, weight, glucose, and lipid changes over time.

**Results:**

51% of black patients and 45% of white patients experienced early study discontinuation (P = .133). Of those who discontinued, significantly more white patients experienced psychiatric worsening (P = .002) while significantly more black patients discontinued for reasons other than efficacy or tolerability (P = .014). Discontinuation for intolerability was not different between groups (P = .320). For the estimated change in PANSS total score over 6 months, there was no significant difference in efficacy between white and black patients (P = .928), nor on the estimated PANSS positive (P = .435), negative (P = .756) or general psychopathology (P = .165) scores. Overall, there was no significant difference in the change in CGI-S score between groups from baseline to endpoint (P = .979). Weight change was not significantly different in white and black patients over 6 months (P = .127). However, mean weight change was significantly greater in black versus white patients at Weeks 12 and 20 only (P = .028 and P = .026, respectively). Additionally, a significantly greater percentage of black patients experienced clinically significant weight gain (≥7%) at anytime compared to white patients (36.1% vs. 30.4%, P = .021). Changes across metabolic parameters (combined fasting and random lipids and glucose) were also not significantly different between groups, with the exception of a greater categorical change in total cholesterol from borderline to high among white subjects and a categorical change from normal to low in high density lipoprotein (HDL) cholesterol among white males.

**Conclusions:**

The findings did not demonstrate overall substantive differences in efficacy or safety between white and black patients diagnosed with schizophrenia or related disorders treated with olanzapine. However, a significantly greater percentage of black patients (36.1%) experienced clinically significant weight gain compared to white patients (30.4%).

## Background

Schizophrenia occurs universally and shows similar patterns of symptoms across populations. The overall prevalence of schizophrenia is estimated to be between 1% to 2% of the population, and the prevalence of major psychotic disorders appears consistent across different ethnic groups [1,2]. In the United States, the incidence of schizophrenia also appears to be uniform across racial and ethnic groups with the exception of higher rates of schizophrenia among racial minorities living in larger cities [2,3]. However, the diagnosis of schizophrenia has been shown to be more frequent in black patients than other ethnic groups [4-7]. Previous studies also suggest that black patients may receive higher doses of antipsychotics [3,8,9], are more likely to receive depot formulation of antipsychotics [9-11], may be less likely to receive a second generation antipsychotic (SGA) [12-15], and have lower medication adherence [15].

Second generation antipsychotics have proven effective in clinical trials and have experienced widespread use for the treatment of schizophrenia. However, there is a great variability in the response profiles of individual patients. Recent research efforts have focused on tailored therapeutics and identifying patients who will have the optimal response with minimal adverse events either before the treatment initiation or early in the course of therapy. The field of genomics, proteomics, and metabolomics are developing rapidly and may offer promise for this purpose. Until then, patient subgroups may be identified at a broader level by baseline characteristics such as metabolic status, duration of illness, symptom patterns, and ethnicity. The interest in race and its influence on treatment outcomes is so great that the National Institutes of Mental Health (NIMH) sponsored an ongoing study, PAARTNERS (Project among African Americans to explore risks for schizophrenia) seeking to identify genetic polymorphisms that confer risk to schizophrenia among black patients [16].

Race may also be an important demographic risk factor for metabolic abnormalities. The incidence of diabetes, dyslipidemias, and obesity are known to be more prevalent among blacks in the general population. This increased risk is also likely to extend to those suffering from mental illness. However, the extent and nature of this risk has yet to be adequately addressed.

To our knowledge, there have been no double-blind, randomized controlled trials designed to compare antipsychotic differences among ethnic groups. The majority of schizophrenia patients enrolled in clinical trials is of white descent and separate results for ethnic minorities are infrequently reported. Currently, minimal information exists to help our understanding of any potential ethnic differences in response and treatment-emergent adverse events (TEAEs). Our study analyzed a large clinical trial dataset of patients treated with olanzapine to compare efficacy and safety characteristics between black and white patients.

## Methods

### Study Design

Data were pooled from 6 similarly designed, randomized, double-blind studies of olanzapine versus other atypical antipsychotics (risperidone, quetiapine, ziprasidone, and aripiprazole) in the treatment of DSM-IV criteria for schizophrenia, schizophreniform disorder, or schizoaffective disorder [17-22]. For our selection criteria, we chose studies based upon treatment duration of no less than 6 months that contained at least one double-blinded treatment arm of oral olanzapine. Eligible patients received olanzapine at doses between 5 to 20 mg. Studies were a mixture of both fixed dosed and flexibly dosed designs. The 6 studies which met these criteria were conducted from 1995 to 2003. Full details of the study designs are reported in the published articles and briefly summarized in Table 1. Each individual study was approved by the Ethics Committee from each participating institution, patient confidentiality was not breached, and the study was done in accordance to the Declaration of Helsinki with written informed consent obtained. We performed a posthoc analysis focused on the treatment of olanzapine in white and black patients.

**Table 1:** Studies Used in Analysis

Study	Population	Duration (weeks)	**Dose (mg/day)**	Inclusion Criteria	Fasting Labs
HGLB^17^	Schizophrenia	28	10-20	(i) PANSS Total ≥75 (ii) PANSS Positive ≥4 (iii) CGI-S ≥4	Yes
HGJU^18^	Schizophrenia, Schizoaffective with Comorbid Depression	24	10-20	(i) MADRS Total ≥16 (ii) MADRS item #2 ≥4	Yes
HGJB^19^	Schizophrenia, Schizoaffective with negative Symptoms	24	10-20	(i) GAF ≤60 (ii) PANSS Negative ≥4 on at least 3 items or ≥5 on at least 2 items	No
HGHJ^20^	Schizophrenia	28	10-20	(i) BPRS ≥42 (ii) PANSS Positive ≥4 (iii) CGI-S ≥4	Yes
HGBG^21^	Schizophrenia, Schizophreniform, Schizoaffective	28	10-20	(i) BPRS ≥42	No
HGGN^22^	Schizophrenia, Schizoaffective	52	5-20	(i) Illness duration ≥2 yrs (ii) PANSS Positive≥4 on 2 items (iii) BPRS ≥18	No

Abbreviations: PANSS = Positive and negative syndrome scale; CGI-S = Clinical global impression of severity; MADRS = Montgomery Asberg depression rating scale; GAF = Global assessment of functioning; BPRS = Brief psychiatric rating scale

### Assessments

Efficacy measures included the change in the Positive and Negative Syndrome Scale (PANSS) total score, PANSS positive, PANSS negative, and PANSS general psychopathology scores over the 24- to 28- Week period. Change in Clinical Global Impression severity (CGI-S) score and time to all-cause discontinuation was also evaluated. Safety measures included reporting of TEAEs, categorical assessment of extrapyramidal symptoms (EPS), which was conducted using the Abnormal Involuntary Movement Scale (AIMS)16, a 12-item scale designed to record the occurrence of dyskinetic movements, and used as the primary measure to assess the incidence of tardive dyskinesia (TD). The Barnes Akathisia Scale 18 was used to assess akathisia at baseline and during treatment. The modified Simpson-Angus Scale was used to measure treatment-emergent parkinsonism. The definitions used for treatment emergent EPS based on the above scales were AIMS: score ≥3 in one or more body regions (Items 1-7) OR score = 2 in two or more body regions (Items 1-7) for at least 1 month; Barnes Akathisia global score of 2 or greater at any postbaseline visit and a baseline score <2; Simpson Angus >3 at any postbaseline visit and baseline score ≤3; AIMS: score ≥3 in one or more body region (Items 1-7) OR score = 2 in two or more body regions (Items 1-7) for at least 1 month. Glucose, lipid, and weight changes were assessed over time. Fasting, random, and combined laboratory outcomes were analyzed as both categorical and continuous measures.

### Statistical Analysis

Data from the 6 studies were pooled for these analyses. Patients were analyzed on an intent-to treat (ITT) basis for all analyses. Baseline characteristics were compared between black and white patients by a Cochran-Mantel Haenszel (CMH) test for categorical variables and by analysis of variance for the continuous variables (both adjusted by study). The proportion of patients who experienced early study discontinuation, TEAEs, and changes in EPS were compared between groups by a CMH test, adjusting for protocol. Time to early discontinuation was assessed by the Kaplan-Meier method and log rank test. Analyses of the change from baseline in efficacy rating scales (PANSS total, PANSS positive, PANSS negative, PANSS general psychopathology, and CGI-S) and weight were performed using a mixed- model repeated measures (MMRM) analysis over 24 weeks. The models included the fixed, categorical, effects of ethnic group, therapy week, baseline rating score (weight), protocol, investigative site, and therapy week by group interaction.

Differences in categorical lipid, glucose, and weight values were compared between groups by a CMH test, adjusting for protocol. Fasting triglyceride levels were categorized as "normal", "borderline", "high", and "extremely high" based on the National Cholesterol Education Project (NCEP) thresholds [23]. Categorical levels of normal (<100 mg/dl), borderline, (≥100 mg/dl to <126 mg/dl) and high (≥126 mg/dl) for fasting glucose are based on American Diabetic Association (ADA) criteria [24]. Changes from baseline to endpoint in lipid and glucose continuous measures were analyzed for within group changes with a Wilcoxon-signed rank test and between group differences were assessed with a ranked analysis of variance (ANCOVA) model, controlling for protocol and baseline value and using the method of last observation carried forward (LOCF).

Patients with a baseline and at least one post-baseline measure were included in all efficacy and laboratory analyses. Adverse event analyses were performed on all patients who took at least one dose of study drug. All P-values were based on two-tailed tests with significance level of .05 and ANCOVA models used Type III sum of squares. To test for the optimal within subject covariance matrix in each MMRM model, the following structures were tested: unstructured, toeplitz, auto regressive, and compound symmetric (both also including the heterogeneous version). The optimal fit was determined by Bayesian Information Criteria (BIC).

## Results

### Patient Characteristics

The pooled baseline demographics, psychiatric history, and disease severity of patients are shown in Table 2. Patients were chronically ill, diagnosed predominately with schizophrenia, and exhibited an average illness duration of about 16 years. Significant differences in baseline characteristics between black and white patients included: a significantly higher percentage of black patients (86.6%) diagnosed with schizophrenia compared to white patients (76.3%, P < .001), a significantly higher percentage of white patients (23.1%) diagnosed with schizoaffective disorder compared to black patients (13.4%, P < .001), a significantly higher percentage of black patients (87.1%) whose geographical region was the United States compared to white patients (69.1%, P < .001), and a statistically significantly higher CGI-S score for white patients (4.60, SD = .78) than black patients (4.42, SD = .69, P = .002).

**Table 2:** Patient Demographics

Variable		Black Descent	White Descent	P-values
		(N = 372)	(N = 602)	
Gender	Male	264 (71.0%)	399 (66.3%)	0.132
	Female	108 (29%)	203 (33.7%)	
Age (yrs)	Mean	39.96	39.79	0.514
	SD	9.48	10.91	
	Median	40.49	39.85	
Weight (kg)	Mean	86.62	84.01	0.257
	SD	21.93	20.73	
	Median	82.43	81.12	
BMI (kg/m2)		N = 362	N = 596	0.587
	Mean	29.06	28.47	
	SD	6.95	6.51	
	Median	28.15	27.33	
Geographic Region	USA	324 (87%)	416 (69.1%)	<0.001
	Europe	4 (1.1%)	107 (17.8%	
	South America	37 (10%)	76 (12.6%)	
	Other	7 (1.9%)	3 (0.5%)	
PANSS Total	Mean	88.15	89.27	0.528
	SD	15.45	17.66	
	Median	87.0	88	
PANSS Positive	Mean	21.31	21.08	
	SD	5.05	5.98	
	Median	21.5	21	
Diagnosis	Schizophrenia	322 (86.6%)	459 (76.3%)	<0.001
	Schizoaffective	50 (13.4%)	139 (23.1%)	<0.001
	Schizophreniform	0 (0%)	4 (0.7%)	0.298
CGI Score	Mean	N = 329	N = 505	0.002
	SD	4.42	4.60	
	Median	0.69	0.78	
		4.00	4.00	
# Previous Episodes		N = 184	N = 341	0.296
	Mean	8.53	7.07	
	SD	7.53	8.98	
	Median	6.0	4.0	
Illness Duration		N = 368	N = 600	0.547
	Mean	16.83	15.92	
	SD	10.38	10.43	
	Median	15.45	15.17	

Abbreviations: N = All randomized patients with at least one post-baseline visit; SD = Standard Deviation; BMI = Body max index; PANSS = Positive and negative syndromes scale; CGI = Clinical global impression

### Patient Disposition

**Figure 1:**
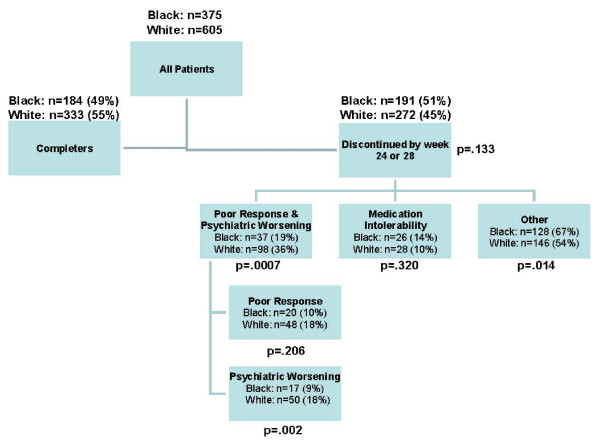
**Patient disposition. **Diagram summarizing patient disposition.

The percentage of patients completing the study and reasons for discontinuation are shown in Figure 1. 51% (191/375) of black patients discontinued treatment and 45% (272/605) of white patients discontinued treatment with olanzapine for any reason over the 24-28 weeks (P = .133). Of those who discontinued, significantly more white patients discontinued due to psychiatric worsening compared to black patients (P = .002) while significantly more black patients discontinued for "other" reasons (e.g., patient decision, physician decision, sponsor decision, noncompliance, lost to follow-up, and criteria not met, P = .014). Discontinuations for medication intolerability were not different between groups (P = .320). The mean modal dose of olanzapine for the black patient group was 15.5 mg (SD = 4.3) and was 15.3 mg (SD = 4.2) for the white patient group. The median exposure was 164 days for black patients and 166 days for white patients.

### Efficacy

**Figure 2:**
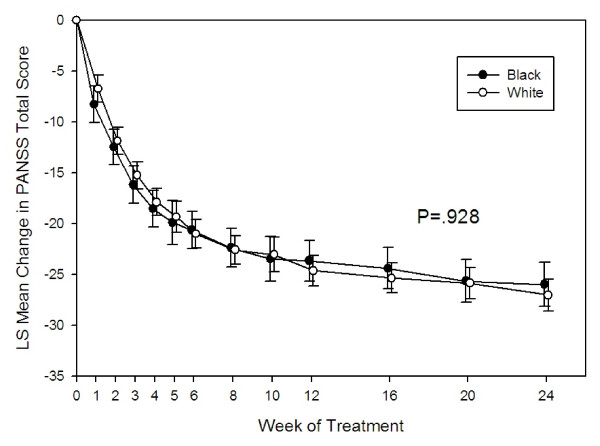
**Estimated change in PANSS total score in olanzapine-treated black and white patients over 24 weeks**. Graph based on MMRM Model including fixed terms baseline PANSS total score, treatment week, protocol, investigator, race, and ethnic origin × treatment week. Race P-value = 0.93.

The primary efficacy measure was the estimated change from baseline in the PANSS total score over 6 months. At the end of treatment, the estimated mean reduction was 27.0 points (SE = .80) for the white patient group and 26.0 (SE = 1.10) for the black patient group. Overall, the reduction in the PANSS total score was not found to be statistically significantly different between groups (P = .928, Figure 2). Likewise, the overall estimated mean changes from baseline in PANSS positive, PANSS negative, and general psychopathology scores were also not statistically different between groups (P = .435, P = .756, P = .165, respectively).

**Figure 3:**
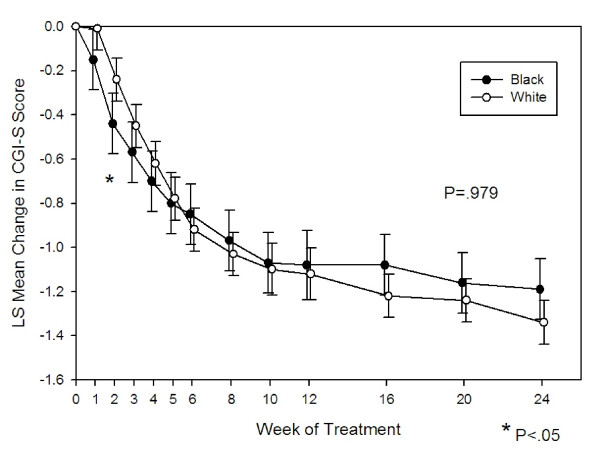
**Estimated change in CGI-Severity score in olanzapine-treated black and white patients over 24 weeks**. A statistically significant difference was found in the effect of race with respect to time (P = 0.027). Black patients showed a more rapid improvement in the first 5 weeks.

For the CGI-S scale, at the end of treatment, the estimated mean reductions were 1.34 points (SE = .05) for the white patient group and 1.19 (SE = .07) for the black patient group. Overall, there was no significant difference in the change in CGI-S score between groups from baseline to endpoint (P = .979). We did find, however, a statistically significant difference in the effect of race with respect to time (P = .027) over the first 5 weeks. As seen in Figure 3, black patients show a more rapid improvement compared to white patients over the first five weeks of treatment with a statistical difference seen at Week 2 (P < .05). No statistically significant difference between groups was seen for the remainder of the analysis.

Given recent attention to the outcome of all-cause time to discontinuation in the treatment of schizophrenia as a proxy measure for treatment effectiveness (primary efficacy measure in the CATIE trial), we performed a similar analysis for this dataset. We found no statistically significant difference in time to all-cause discontinuation between black and white patients (median time: 24 weeks versus not estimatable, respectively; P = .078).

### Safety

The frequency of TEAEs occurring at ≥5% or any statistically significant events between groups are shown in Table 3. The highest reported adverse events in both groups were somnolence, headache, and weight increased. Adverse events reported statistically significantly more often in the white patient group compared to the black patient group included: insomnia, tremor, disturbance in attention, irritability, chills, and initial insomnia. Adverse events reported statistically significantly more often in the black patient group compared to the white patient group included increased weight, migraine, cyst, left ventricular hypertrophy, and leukopenia.

**Table 3:** Frequency of TEAEs Occurring at ≥5% or Any Statistically Significant Events

Event Term	White N = 600 N (%)	Black N = 369 N (%)	Total N = 969 N (%)	p-value *	P = value **
Somnolence	88 (14.7)	52 (14.1)	140 (14.4)	0.851	0.792
Headache	76 (12.7)	62 (16.8)	138 (14.2)	0.088	0.057
Weight Increased	72 (12.0)	62 (16.8)	134 (13.8)	0.044	0.031
Insomnia	64 (10.7)	26 (7.0)	90 (9.3)	0.068	0.050
Anxiety	61 (10.2)	23 (6.2)	84 (8.7)	0.035	0.216
Dry Mouth	57 (9.5)	33 (8.9)	90 (9.3)	0.820	0.785
Nausea	47 (7.8)	27 (7.3)	74 (7.6)	0.804	0.858
Sedation	47 (7.8)	22 (6.0)	69 (7.1)	0.305	0.223
Increased Appetite	41 (6.8)	32 (8.7)	73 (7.5)	0.317	0.542
Depression	40 (6.7)	19 (5.1)	59 (6.1)	0.407	0.369
Fatigue	38 (6.3)	19 (5.1)	57 (5.9)	0.485	0.205
Neopharyngitits	36 (6.0)	15 (4.1)	51 (5.3)	0.236	0.165
Vomiting	35 (5.8)	13 (3.5)	48 (5.0)	0.127	0.154
Dizziness	32 (5.3)	20 (5.4)	52 (5.4)	1.000	0.791
Akathesia	31 (5.2)	11 (3.0)	42 (4.3)	0.143	0.230
Diarrhoea	31 (5.2)	24 (6.5)	55 (5.7)	0.394	0.375
Tremor	31 (5.2)	7 (1.9)	38 (3.9)	0.010	0.019
Disturbance in Attention	13 (2.2)	1 (0.3)	14 (1.4)	0.023	0.028
Irritability	12 (2.0)	0 (0.0)	12 (1.2)	0.005	0.012
Chills	8 (1.3)	0 (0.0)	8 (0.8)	0.027	0.032
Initial Insomnia	7 (1.2)	0 (0.0)	7 (0.7)	0.049	0.046
Migraine	1 (0.2)	4 (1.1)	5 (0.5)	0.073	0.033
Cyst	0 (0.0)	3 (0.8)	3 (0.3)	0.055	0.022
Left Ventricular Hypertrophy	0 (0.0)	2 (0.5)	2 (0.2)	0.145	0.017
Leukopenia	0 (0.0)	2 (0.5)	2 (0.2)	0.145	0.006

*Fisher Exact Test; **CMH Test adjusted for protocol

Abbreviation: N = number of randomized patients who have taken at least 1 dose of study drug and have a post-baseline visit; TEAEs = treatment-emergent adverse events

No significant differences were found between black and white patients who experienced categorical changes at anytime on the Barnes Akasthisia Scale (a global score ≥2; P = .226), the modified Simpson-Angus Scale (a total score >3; P = .071), or AIMS (a score of ≥3 on one or more body regions or a score of 2 in two or more body regions for at least one month; P = 0.116).

**Figure 4:**
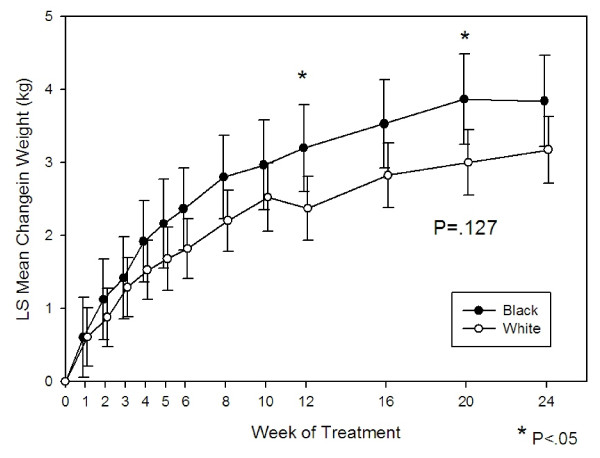
**Estimated change in weight in olanzapine-treated black and white patients over 24 weeks**. Graph based on MMRM Model including fixed terms baseline weight, treatment week, protocol, investigator, race, and ethnic origin × treatment week. Race P-value = 0.13.

Baseline data for weight and body mass index (BMI) was not significantly different for black or white patients (P = .257 and P = .587, respectively). The fixed effect term for the estimated overall change from baseline over the 24 weeks was not found to be statistically significantly different between the two groups (P = .127, Figure 4). However, we found a statistically significant greater increase in weight change in black patients compared to white patients at Weeks 12 and 20 (Week 12: .83 kg, SE = .38, p = .028; Week 20: .87 kg, SE = .39, P = .026). Additionally, the percentage of patients experiencing clinically significant weight gain (defined as an increase from baseline ≥7% at anytime) was 36.1% in the black patient group compared to 30.4% in the white patient group (P = .021)

**Figure 5:**
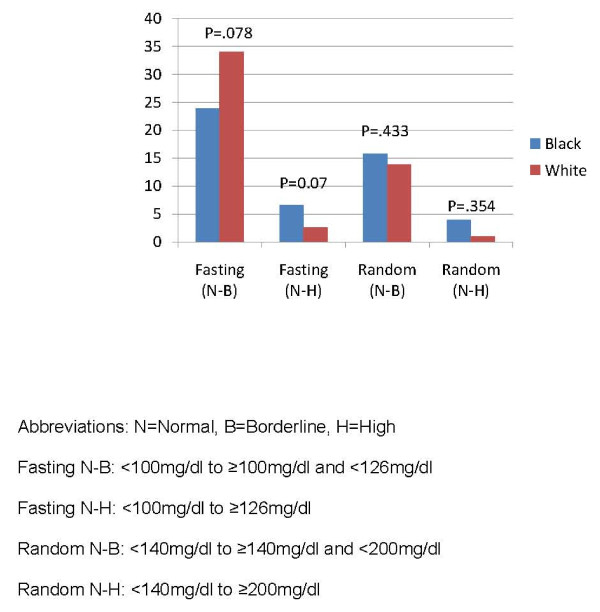
**Percentage of patients experiencing adverse categorical glucose changes at anytime**.

Since one-half (3 of 6) of the studies required the collection of blood samples in the fasting state, glucose changes are reported separately as "fasting", "random" (the 3 non-fasting studies) or "fasting and random combined", where we pooled the three non-fasting studies, and the three fasting studies together. The mean change from baseline to endpoint over 24 weeks in both the fasting only studies and random only studies was not significantly different between black and white patients (P = .245 and .557, respectively). The within group mean change in glucose from baseline in the fasting only studies was 2.30 mg/dl (SD = 37.1, P = .689) in the black group and 3.91 mg/dl in the white group (SD = 28.4, p = .033). The within group mean change in glucose from baseline in the random only studies was 9.02 mg/dl (SD = 35.5, P = .179) in the black group and 4.75 mg/dl (SD = 38.1, P = .067) in the white group. We found no significant difference between groups on the percentage of patients that experienced an adverse categorical change in glucose levels at anytime during the study (Figure 5). We also looked at the occurrence of treatment-emergent diabetes (terms "Diabetes mellitus" or "Type 2 diabetes mellitus") and found no statistically significant difference between groups (4 white patients and 4 black patients, 0.7% versus 1.1%, P = 0.532).

**Figure 6:**
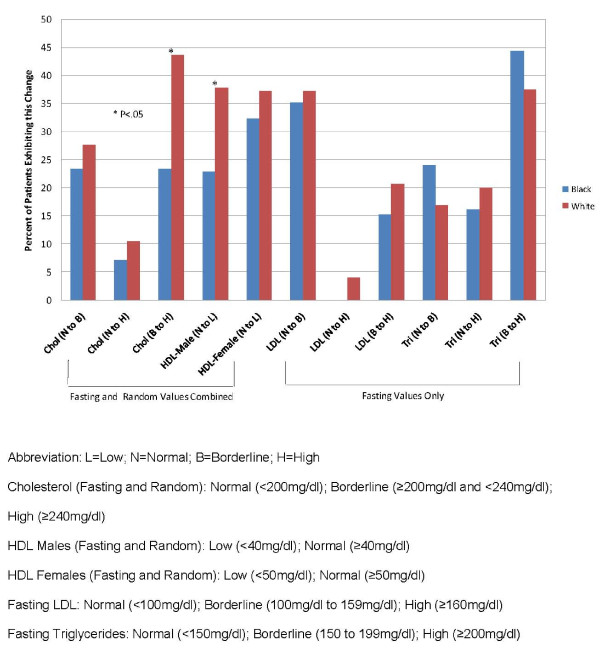
**Treatment-emergent categorical lipid changes**.

Categorical changes in lipids are also reported separately based upon protocol-specified fasting collection methods. Fasting and random values were combined across all six studies for total cholesterol and high-density lipoprotein (HDL) cholesterol while fasting only studies are reported for low density lipoprotein (LDL) cholesterol and triglycerides. No statistically significant differences were seen in any fasting values for LDL or triglycerides between patients of either group (See Figure 6). However, significantly more white patients (59/135) had a categorical increase in fasting and random cholesterol levels from borderline (≥200 mg/dl and <240 mg/dl) to high (≥240 mg/dl) compared to black patients (15/64; P = .019). Additionally, significantly more white males (33/87) showed a decrease in HDL cholesterol levels from normal (≥40 mg/dl) to low (<40 mg/dl) compared to black males (24/105; P = .039).

## Discussion

The results of this post-hoc analysis suggest similar efficacy response for black and white patients with schizophrenia treated with olanzapine. The reduction on PANSS scores and PANSS subscale scores were similar for both groups. We also found no overall differences on changes in the CGI score and all-cause time to discontinuation. Our data is consistent with other studies that did not show a difference between black and white patients treated with antipsychotics [25,26]. The CATIE (Clinical Antipsychotic Trials of Intervention Effectiveness) study [27] recently reported preliminary results on the effect of ethnicity in the treatment of schizophrenia [25]. They reported no difference in all-cause time to discontinuation and PANSS Total score between white, black, and Hispanic subgroups. Another study looked at the impact of race on the efficacy and safety of long-acting risperidone compared to placebo [26]. These results showed there was no effect of race on the improvement of PANSS total scores from baseline to endpoint. In contrast, one study conducted in Africa did show a greater reduction in PANSS total score by mixed descent and black patients compared to white patients [28]. While one potential explanation of this finding could be described by significant differences in baseline PANSS scores, the divergence to our results and other studies is worth noting.

As overall discontinuation rates did not differ between groups, significantly more white patients (18%) discontinued due to psychiatric worsening than black patients (9%). While the number of patients in this sample was small, this may warrant further discussion. Since PANSS total improvements were similar between groups, the discontinuation due to psychiatric worsening in the white subgroup is counter-intuitive. This may be explained by the variability in individual responses that may not be captured in our larger dataset and analysis methods. The largest percentage of discontinuations from the studies was due to reasons other than poor response/psychiatric worsening and medication intolerability. In fact, a higher percentage of black patients discontinued from the study compared to white patients due to these other reasons (patient decision, physician decision, sponsor decision, noncompliance, lost to follow-up, and criteria not met). This is fairly consistent with the overall CATIE study results, where the largest percentages of treatment discontinuations were due to patient decision. A better understanding as to how these other reasons effect individual treatment outcome may give additional insight into the treatment approach to schizophrenia.

A recent study suggested there may be a higher predictability of weight gain with clozapine in black patients [29]. In our analysis, a statistically significant difference in weight change was found in the black patient group compared to the white patient group only at Weeks 12 and 20, but the numerical difference between the two groups was less than 2 pounds. Overall baseline to endpoint changes in weight was not statistically significant between groups. However, a statistically higher percentage of black patients (16.8%) versus white patients (12.0%) reported "increased weight gain" as a treatment emergent adverse event. Likewise, a statistically significantly greater number of black patients reported categorical clinically significant weight gain of ≥7% anytime during the study. Therefore, while overall mean weight change throughout the studies was not different, there is some evidence in our study to suggest that black patients may be at greater risk for weight changes.

Research has also suggested that glucose, lipid, and overall metabolic abnormalities may be higher with second generation antipsychotics in black patients [30-33]. The CATIE study showed that, at baseline, black patients had a higher incidence of dyslipidemias. Other studies have evaluated the ethnic differences with medication on visceral adiposity, insulin resistance, glucose, and cholesterol [34-36]. These studies concluded that ethnic minorities may have a greater risk of treatment-emergent metabolic adverse events which may differ depending on the medication prescribed. A recent review article looking at ethnic differences in the risks of adverse events in treating psychoses and depression showed a higher relative risk for hyperglycemia in black patients compared to non-black patients and a higher relative risk for diabetes mellitus in non-white patients compared to white patients. [37]. Unlike our analysis, this study included patients with depression. In our study, no significant differences were seen between groups in both fasting and random glucose concentrations, as well as treatment emergent diabetes. There was, however, a statistically significant within-group mean change in fasting glucose concentrations in the white subgroup. Within group differences were not seen in the black subgroup. Additional long-term data are needed to better understand the potential effect of race on glucose levels with medication treatment.

For lipids, we also did not find consistent differences between groups, with a couple of exceptions. In the combined fasting and random cholesterol analysis, categorical change from borderline to high total cholesterol was greater in the white subgroup compared to the black subgroup. Combined fasting and random HDL cholesterol changes from normal to low in the white male patients was also statistically significant compared to black male patients. These statistical changes for the white subgroup, although a result of combined random and fasting blood testing, differ from results of other studies. [34-36].

Our study did not show any significant differences between groups on measures of EPS. Earlier studies have identified race as a risk factor for the development of TD and pointed that race may be a factor in predicting a poor course of TD. This is in contrast to the CATIE trial analysis, where white patients had worse outcomes on the Simpson-Angus Scale (P <.001), and Barnes Akathisia Scale (P < .001) compared to the black patient group. More data may be needed to confirm the potential effect of race on EPS and TD outcomes.

While we did not find significant differences in overall outcomes between black and white patients, the importance of individual variability in antipsychotic efficacy and tolerability must be considered. We did not look at genetic differences; however, there is a growing body of research on genetic susceptibility to schizophrenia, medication treatment, and the potential effect of race. This data thus far have been fairly inconclusive [38-41]. A recent candidate gene association analysis with risperidone showed a potential genetic link to poor response in white patients versus black patients, but sample size was small and requires replication [42]. Patients with schizophrenia are a highly diverse population and a clinician must be sensitive to the individual differences that exist and may not be seen in a dataset such as ours.

One important limitation to this study is that this was a pooled, posthoc analysis, and therefore, it was not prospectively powered to specifically assess efficacy comparisons between black and white patients treated with schizophrenia However, we attempted to overcome this limitation by combining six similarly designed studies and producing a large dataset for analysis. In addition, the results are adjusted for the six separate studies with "protocol" used as a term in the MMRM model. The total number of black patients in this clinical trial dataset is impressive given the small amount of published information on the potential effect of race on treatment outcomes in schizophrenia. While the CATIE study had a large number of black patients (n = 513 across all treatment groups) in their recent analysis looking at ethnic variability in response and adverse events to antipsychotics, our dataset for olanzapine-treated black patients (n = 375) is the largest for a single drug treatment, to our knowledge.

Another limitation is the focus on only patients of black descent, primarily from the United States. Due to the small number of patients in our studies representing Hispanic, Asian, and other minorities, comparisons among these ethnic subgroups were not possible, but are also needed in the literature.

Within our analysis, there is a lack of information on prior exposure to atypical antipsychotics. Since these were comparative trials and patients suffered from chronic schizophrenia, it is likely that there were prior medication exposures which could affect their baseline values on weight and glucose. Therefore, our results must be interpreted with respect to prior exposures.

There was a high drop-out rate in our analysis with almost half of patients discontinuing the study prior to 24-28 weeks. To account for this in the analysis, we used MMRM analysis for efficacy (PANNS and CGI-S) and weight. Last observation carried forward was used to analyze lipids, glucose, EPS, and weight. MMRM could not be performed on lipids, glucose, and EPS since these measures were not collected at every visit in each study included in the analysis.

We also performed many comparisons in this analysis and did no type of formal statistical adjustment for this fact. For example, while black patients had a statistically significantly greater baseline CGI-S score than white patients, we felt this difference was not clinically significant to warrant a statistical adjustment. For the efficacy endpoints, we choose only a selected few measures that we felt were clinically significant. For the safety measures, we ignored multiplicity as a conservative measure in order to evaluate any potential safety signals which may be missed by lowering the P-value.

## Conclusion

The present analysis suggests that olanzapine is equally efficacious in black and white patients in the treatment of schizophrenia. Prospective studies are necessary to confirm if black and white patients may have different response patterns to antipsychotics, which would help clinicians tailor antipsychotic therapies accordingly.

## Competing interests

VLS, KWP, JG, SKW, RC, VPH, and TD are employees of Lilly USA, LLC, a subsidiary of Eli Lilly and Company.

## Authors' contributions

VLS conceived the study and contributed to the design and coordination. JLS and JG performed the statistical analysis. KWP wrote the initial and subsequent drafts of the manuscript. SKW coordinated the development of the initial and final drafts. All authors participated in the analysis and interpretation of the data, and revising the manuscript for critically important intellectual content. In addition, all authors read and approved the final version of the manuscript.

## Pre-publication history

The pre-publication history for this paper can be accessed here:

http://www.biomedcentral.com/1471-244X/10/89/prepub
